# Uso Apropriado das Novas Diretrizes de Função Diastólica na Avaliação de Atletas. Nem Sempre é o que Parece Ser

**DOI:** 10.36660/abc.20190689

**Published:** 2020-07-28

**Authors:** Carlos Eduardo Suaide Silva

**Affiliations:** 1 Diagnósticos da América SA São PauloSP Brasil Diagnósticos da América SA – Cardiologia, São Paulo, SP - Brasil

**Keywords:** Disfunção Ventricular, Diagnóstico por Imagem, Ecocardiografia/métodos, Diretrizes, Esportes, Exercício, Atletas

A correta avaliação da função diastólica pela ecocardiografia tem sido sempre um grande desafio para os cardiologistas que trabalham com o método. Em parte, pelas mudanças frequentes nas diretrr izes decorrentes da grande quantidade de novas informações a respeito de tão complexo assunto, em parte por certa confusão gerada pelas próprias diretrizes, muitas vezes contraditórias ou pouco explicativas.^1^ Entretanto, estamos caminhando para um maior entendimento do que ocorre nessa importante fase do ciclo cardíaco. A última diretriz de função diastólica publicada em 2016, apesar de ainda apresentar algumas incoerências e nos deixar com algumas dúvidas em determinadas situações clínicas, veio esclarecer diversos pontos e corrigiu algumas distorções da diretriz anterior.^2^

Como sempre acontece na medicina, toda vez que temos alguma mudança de paradigma, ou uma nova entidade clínica é descoberta, passamos inicialmente por uma fase de exagero no diagnóstico seguida por uma fase de descrédito para finalmente atingirmos o equilíbrio com a maturidade e o conhecimento adquirido com o tempo. Foi assim com o prolapso da valva mitral, que apresentava incidência de mais de 35% em mulheres jovens no início dos anos 1970 e que hoje sabemos ser de aproximadamente 2,4% sem diferença entre os sexos.^3^ O mesmo ocorreu com o diagnóstico de não-compactação ventricular e diversas outras entidades clínicas e, porque não dizer, com o diagnóstico de disfunção diastólica. Quantos idosos absolutamente saudáveis não foram diagnosticados com disfunção diastólica leve (grau I) por apresentar apenas inversão da relação E/A no fluxo mitral ao Doppler? Almeida et al.,^4^ verificaram o impacto da utilização da diretriz de 2009 em relação à de 2016 no diagnóstico de disfunção diastólica nessa população (1000 indivíduos com mais de 45 anos) e encontraram apenas 1,4% de disfunção diastólica onde haveria 38,2% usando a diretriz anterior.

Dessa forma, com essa nova diretriz parece que chegamos a esse equilíbrio e com a aplicação correta de seus critérios diminuímos significativamente esse exagero no diagnóstico de disfunção diastólica, principalmente na população idosa. Entretanto, talvez ainda deixemos de fazer esse diagnóstico, felizmente em número bem menor de casos, em outras situações clínicas. Particularmente em atletas, a função diastólica precisa ser avaliada com mais atenção.

O exercício é um forte estímulo para a adaptação muscular e há bastante evidências que comprovam que o mesmo é responsável por mudanças na forma e no débito cardíacos.^5^

As adaptações impostas ao coração dependem, evidentemente, do tipo de exercício realizado. Assim, didaticamente falando, atletas que realizam exercícios de resistência (dinâmicos) e que trabalham em altas frequências cardíacas, como os maratonistas ou nadadores, sofrem adaptações diferentes daqueles que fazem exercícios isométricos (estáticos) onde a frequência cardíaca é mais baixa e há predominante aumento da pressão arterial, como ocorre com halterofilistas. Na prática, grande parte dos exercícios são mistos como ocorre com ciclistas e remadores, por exemplo.

**Figura 1:**
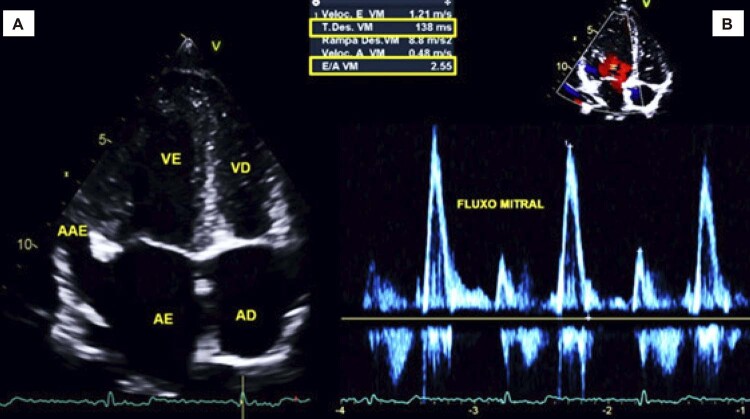
– *À esquerda corte apical de 4 câmaras ao eco bidimensional. À direta padrão do fluxo mitral ao Doppler em atleta jovem. AAE = apêndice atrial esquerdo; AD: átrio direito; AE: átrio esquerdo; E/A VM: relação E/A da valva mitral; T.Des. VM: tempo de desaceleração da onda E do fluxo mitral; VD: ventrículo direito; VE: ventrículo esquerdo.*

No primeiro grupo (maratonistas), onde o débito cardíaco pode chegar a até dez vezes o valor de repouso, o coração precisa se adaptar de diversas maneiras, seja partindo de uma frequência cardíaca basal muito baixa (bradicardia), seja aumentando seu volume sistólico (hipertrofia excêntrica), seja tornando mais efetiva a sua função de bomba extraindo o máximo de suas funções sistólica e diastólica. A diástole desses atletas precisa ser extremamente eficiente porque em alta frequência cardíaca ela se encurta e o coração tem pouco tempo para se encher. Por isso, assim que a valva mitral se abre o ventrículo esquerdo precisa se encher rapidamente, apresentar um relaxamento extremamente eficaz e “sugar” a maior quantidade de sangue possível para gerar uma sístole efetiva. Isso explica a ampla onda E do fluxo mitral ao Doppler seguida de uma pequena onda A (pois sobra pouco sangue para entrar no ventrículo na telediástole) gerando um padrão de fluxo semelhante em morfologia ao padrão restritivo, mas que reflete, na realidade, uma diástole supranormal^6^ ( Figura 1 ).

No segundo grupo (halterofilistas) onde o coração é submetido a altas pressões, sem grande aumento da frequência cardíaca, observamos um predominante aumento da espessura miocárdica, sem dilatação (hipertrofia concêntrica, rigidez aumentada e tempo de relaxamento prolongado levando ao aumento do tempo de desaceleração da onda E e invertendo a relação E/A do fluxo mitral) .

Essas situações são extremas e os exemplos didáticos, mas na realidade, a avaliação da função diastólica em atletas é muitas vezes bem mais complexa do que isso. Vamos mostrar através de dois exemplos clínicos como a utilização racional das novas diretrizes associada às técnicas avançadas de ecocardiografia e à história clínica dos pacientes podem chegar a um diagnóstico correto e mais refinado da função diastólica nessa população.

**Figura 2:**
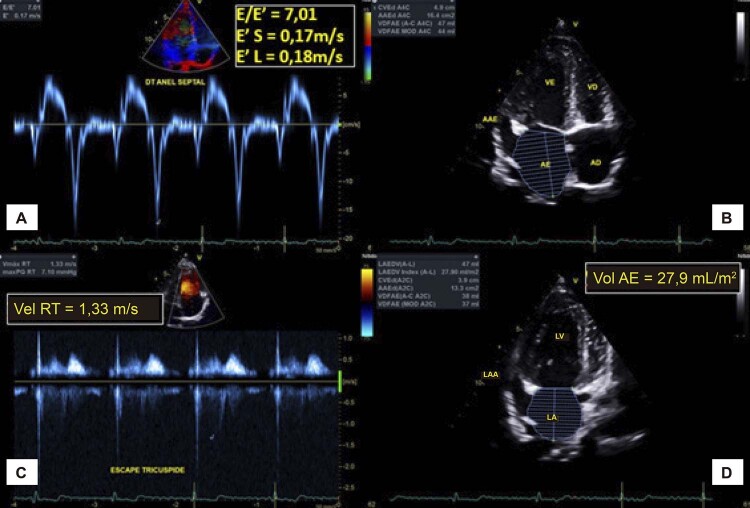
– *Doppler tecidual do anel mitral (superior, esquerda); corte apical de 4 câmaras ao eco bidimensional (superior direita); curva do Doppler contínuo do refluxo tricúspide (inferior esquerda); corte apical de 2 câmaras ao eco bidimensional (inferior direita). AAE: apêndice atrial esquerdo; AD: átrio direito; AE: átrio esquerdo; E’L: velocidade da onda e’ lateral; E’S: velocidade da onda e’ septal; VD: ventrículo direito; VE: ventrículo esquerdo; Vol AE: volume indexado do átrio esquerdo.*

Exemplo 1: masculino, 16 anos, jogador de futebol (o mesmo paciente da Figura 1 ). Analisando somente o padrão de fluxo mitral desse atleta temos uma relação E/A de 2,25 e um tempo de desaceleração da onda E (TDE) de 138ms, o que caracterizaria um padrão de fluxo mitral do tipo restritivo, não compatível com a clínica de um jovem esportista. Prosseguindo a investigação, observamos ao Doppler tecidual o valor da onda e’ septal de 0,17m/s e da onda e’ lateral de 0,18m/s. A relação E/e’ foi de 7,01 a velocidade do refluxo tricúspide de 1,33m/s e o volume indexado do átrio esquerdo de 27,9ml/m^2^ ( Figura 2 ). Todas as medidas dentro da normalidade, configurando então um padrão de fluxo mitral do tipo supranormal, frequentemente encontrado em jovens e atletas.

**Figura 3:**
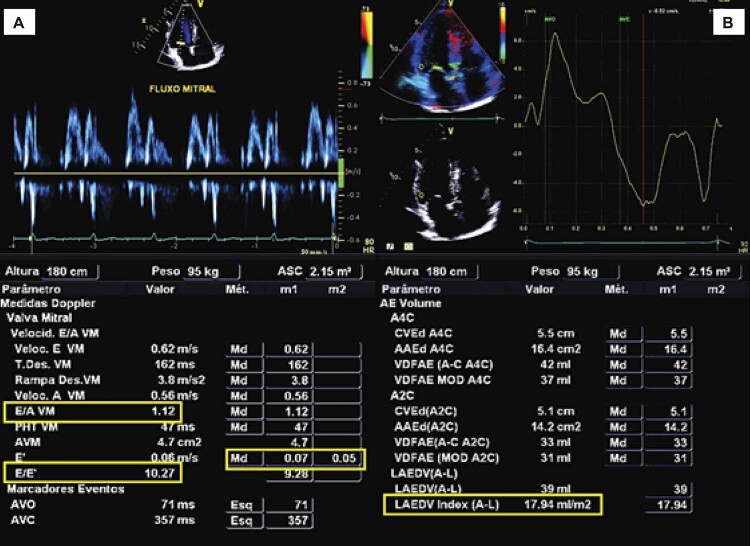
– *Curva do fluxo mitral ao Doppler pulsátil (superior esquerda); curva do Doppler tecidual do anel mitral lateral (superior direita). E/A VM: relação E/A da valva mitral; LAEDVindex(A-L): volume indexado do átrio esquerdo.*

Exemplo 2: masculino, 48 anos, fisiculturista e corredor. O estudo inicial com ecocardiograma tridimensional não apresentou alteração anatômica significativa. Na avaliação da função diastólica observou-se relação E/A de 1,12, velocidades de e’ septal e lateral estimadas em 0,05m/s e 0,07m/s, respectivamente, relação E/e’ de 10,3, volume indexado do átrio esquerdo de 17,9ml/m^2^ e velocidade do refluxo tricúspide de 2m/s ( Figura 3 ). Avaliando a função diastólica desse paciente segundo as diretrizes de 2016, dos quatro critérios maiores, apenas um encontra-se fora da normalidade (velocidades do anel mitral ao Doppler tecidual), o que classificaria a função diastólica como normal.

**Figura 4:**
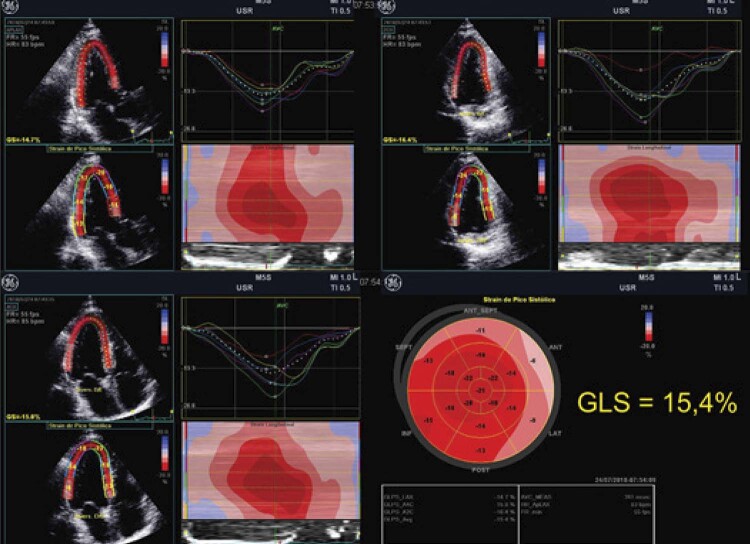
– *Quantificação da deformação miocárdica pela técnica do speckle tracking. GLS: strain longitudinal global.*

Entretanto, chama à atenção os valores alterados das velocidades do anel mitral em um atleta assintomático. Aprofundando a anamnese, o paciente relatou que fazia uso constante de esteroides anabolizantes (propionato de testosterona 30mg, fempropionato de testosterona 60mg, isocaproato de testosterona 60mg, decanoato de testosterona 100mg - Durateston®). Realizando então o estudo da deformação miocárdica pela técnica do *speckle tracking* verificou-se diminuição do *strain* longitudinal global (-15,4%) como mostra a figura 4 . Essa informação modifica totalmente a análise da função diastólica nesse paciente. O fato de ter disfunção sistólica documentada pelo *speckle tracking* direciona a investigação para o segundo algoritmo das diretrizes de 2016 (Pacientes com FE diminuída e pacientes com doença miocárdica e FE normal, após considerar dados clínicos e ecocardiográficos). Um valor do *strain* tão baixo nos leva a pensar em algum grau de disfunção miocárdica em decorrência do uso de esteroides, comprometendo as funções sistólica e diastólica. Segundo as novas diretrizes, não devemos ter disfunção sistólica sem, ao menos, um grau de disfunção diastólica presente pela intrincada relação entre elas. Esse não é um conceito novo. Já em 2008, Lester et al.,^7^ ressaltavam que, “pelo o fato dos parâmetros ecocardiográficos que avaliam a função diastólica serem derivados do Doppler, e os que avaliam a função sistólica serem derivados do estudo bidimensional podemos criar a ilusão de que é possível ter disfunção diastólica isolada”. Portanto, de acordo com as diretrizes de 2016, ao invés de função diastólica normal, diagnosticamos a presença de disfunção diastólica leve nesse atleta.

A população de atletas competitivos e pessoas altamente ativas está em franco crescimento.^8^ Relatos recentes extrapolam as causas de remodelamento cardíaco induzido pelo exercício para além da estrutura ventricular passando a incluir alterações da função diastólica,^9^ da morfologia do ventrículo direito^8^ e estrutura do átrio esquerdo.^10 - 12^

Todas as formas de exercício físico vigoroso, seja em atletas profissionais ou pessoas altamente ativas, envolvem alguma combinação de exercício estático e dinâmico. Os termos estático e dinâmico se referem ao padrão de atividade muscular esquelética e suas consequências no sistema cardiovascular. A atividade estática é caracterizada por contrações curtas e vigorosas de determinados grupos musculares. Durante eventos de atividade estática pura (ou predominante), como ocorre no levantamento ou arremesso de peso, observa-se aumento agudo na resistência vascular e na pressão arterial. O principal papel do sistema cardiovascular nesses atletas é conseguir manter o débito cardíaco frente ao súbito e exagerado aumento da pós-carga. Em contraste, os exercícios dinâmicos (de “endurance”) são caracterizados por contrações e relaxamentos repetitivos, geralmente rítmicos, de grandes grupos musculares o que requer aumento no metabolismo oxidativo. A intensidade da atividade dinâmica pode ser quantificada pelo consumo de oxigênio (VO_2_). A resposta primária do sistema cardiovascular ao exercício dinâmico é aumentar o débito cardíaco para assegurar a chegada de nutrientes ao leito muscular em atividade. O aumento do débito é conseguido pelo aumento do volume sistólico e da frequência cardíaca e da diminuição da resistência vascular periférica.

A função diastólica nessa população de atletas profissionais ou pessoas altamente ativas deve ser normal ou aumentada e qualquer evidência de disfunção diastólica deve nos levar a pensar em patologia.^13^ Dados de uma grande meta-análise sugerem que o exercício físico promove um aumento da função diastólica em atletas por uma combinação de relaxamento proto-diastólico mais efetivo e aumento da complacência ventricular.^14^ O tipo de atividade física também está relacionado às alterações observadas na função diastólica de atletas. O exercício dinâmico leva a um relaxamento ventricular mais efetivo, além da dilatação biventricular, enquanto o exercício estático pode estar relacionado a um certo grau de comprometimento da função diastólica,^15^ geralmente acompanhado de aumento da espessura miocárdica e de hipertrofia concêntrica do ventrículo esquerdo.

Por isso, é fundamental que na avaliação da função ventricular dos atletas, profissionais, amadores ou “de finais de semana”, utilizemos todas as ferramentas disponíveis no arsenal da ecocardiografia. Sempre que possível a fração de ejeção deve ser avaliada pela ecocardiografia tridimensional e a análise criteriosa da deformação miocárdica ( *strain* ) deve sempre ser realizada pela técnica do *speckle tracking* , assim como avaliação cuidadosa da função diastólica seguindo as últimas diretrizes. O *strain* é capaz de detectar alterações incipientes da função sistólica muito antes que ocorra qualquer alteração da contratilidade ao estudo bidimensional ou diminuição da fração de ejeção.

A avaliação de rotina da deformação miocárdica permite a detecção de algum comprometimento miocárdico subjacente nessa população. Além disso, uma análise detalhada da função diastólica deve ser realizada seguindo as últimas diretrizes.

É muito frequente observarmos atletas fazendo uso de fórmulas e esteroides anabolizantes sem qualquer indicação ou acompanhamento médico e um exame ecocardiográfico completo pode detectar precocemente a deterioração da função ventricular, sistólica ou diastólica, e permitir um tratamento adequado evitando maiores danos ao miocárdio.
