# Supplementary material for: Uso Apropriado das Novas Diretrizes de Função Diastólica na Avaliação de Atletas. Nem Sempre é o que Parece Ser
Source: Arq Bras Cardiol. 2020 Jul 28;115(1):134–8. [Article in Portuguese] doi: 10.36660/abc.20190689 (PMC8384328; doi:10.36660/abc.20190689)
Supplement: Translation

# Translation: Appropriate Use of Diastolic Function Guideline When Evaluating Athletes: It is not Always what it Seems to Be

**Authors:** Carlos Eduardo Suaide Silva

**Affiliations:** 1 Brazil Diagnósticos da América SA – Cardiologia, São Paulo, SP - Brazil

**Keywords:** Ventricular Dysfunction, diagnostic Imaging, Echocardiography/methods, Guidelines as Topic, Sports, Exercise, Athletes

The complete echocardiographic evaluation of the diastolic function has always been a great challenge for cardiologists and sonographers, either because of frequent changes found in every new guideline, due to a great amount of recent information about this complex subject, or because of some confusion created by the guidelines themselves, which are many times contradictory or not explanatory.^1^ However, we are moving towards a better understanding of what really happens on this important phase of the cardiac cycle. The last published guideline of 2016, despite presenting some inconsistencies and still not making certain situations clear, clarified several points and corrected some distortions of the previous one.^2^

As usual in Medicine, every time we have a paradigm shift or a new clinical entity appears, we pass first through a moment of overdiagnosis followed by some discredit to finally reach a balance with the maturity and the knowledge acquired over time. The same happened with mitral valve prolapse, which showed an incidence of more than 30% in young women in the early 1970s, but nowadays it is known of about 2.5% in both sexes.^3^ Ventricular non-compaction and many other diseases followed the same pattern, as well as diastolic disfunction. How many healthy elderly people were diagnosed with mild diastolic disfunction (or grade I) due to presence of E/A inversion on spectral Doppler of the mitral inflow? Almeida et al.^4^ tested the impact of using the 2009 guidelines against 2016 for diagnosing diastolic disfunction in 1,000 elderly individuals (over 45 y/o). They found that only 1.4% had some degree of diastolic disfunction according to the 2016 guideline. On the other hand, if the 2009 guideline had been used, this number would rise to 38.2%.

Thus, with this new guideline, we seem to have come to this balance and by using the correct application of its criteria we can significantly reduce the excessive diagnosis of diastolic disfunction, mainly in the elderly. However, we may still fail to diagnose, fortunately in a much lower amount of cases, in another clinical situations. Particularly in athletes, diastolic function needs to be evaluated more carefully.

Exercise is a strong stimulus for muscle adaptation, and there is plenty of evidence proving that it is responsible for changes in shape and cardiac output.^5^

The adaptations imposed to the heart depend, of course, on the type of performed exercise. Therefore, didactically, athletes that perform dynamic exercises and work out at a high heart rate, such as marathon runners or swimmers, suffer different adaptations from those who perform isometric (static) exercises, in which the heart rate is lower and there is predominant increase in blood pressure as it happens with bodybuilders. In practice, most exercises are mixed as in cyclists and rowers, for example.

In the first group (marathon runners), in which the cardiac output can reach ten times the resting value, the heart needs to adapt in a variety of manners, whether starting from a very low basal heart rate (bradycardia), increasing left ventricular volume (eccentric hypertrophy) or even making its pump more effective, by extracting the maximum of its diastolic and systolic functions. The diastole of these athletes must be extremely efficient because tachycardia shortens this phase and the heart has much less time to be filled. Thus, as soon as the mitral valve opens, the left ventricle must fill up quickly, show an extremely effective relaxation and “suck” as much blood as possible to generate an effective systole. It explains the big E wave on mitral Doppler followed by a small A wave (because there is little blood left to fill the ventricle on the end of diastole), thus showing a flow pattern similar to restrictive, but reflecting, in fact, a supernormal diastole^6^ ( Figure 1 ).

**Figure 1:** – *Left: Apical four-chamber view on 2D echo. Right: Doppler mitral flow on a young athlete. LAA: left atrial appendage; RA: right atrium; LA: left atrium; E/A VM: mitral valve E/A ratio; T.Des. VM: mitral valve deceleration; RV: right ventricle; LV: left ventricle..*

In the second group (bodybuilders), in which the heart is subjected to high pressures without increasing its frequency too much, we can find predominant increase in wall thickness without dilatation (concentric hypertrophy, augmented muscle stiffness, and longer relaxation period leading to a prolonged deceleration time of mitral E wave and an inverted E/A ratio).

These situations are exaggerations, and the examples used herein are only for better understanding, but in the real world, diastolic evaluation in athletes is usually much more complex. We will show, with two clinical examples, how the rational use of the new guideline along with advanced echocardiographic techniques and clinical history can lead to a correct and refined analysis of diastolic function on this population.

Example 1: 16-year-old male soccer player (same patient of Figure 1 ). If we look only for the mitral flow pattern of this athlete, we will find an E/A ratio of 2.25 and an E wave deceleration time of 138 ms, which could resemble a restrictive pattern that is incompatible with the status of a young athlete. Tissue Doppler velocity shows septal e’ of 0.17 m/s and lateral e’ of 0.18 m/s. It leads to an E/e’ ratio of 7.01. Tricuspid regurgitation velocity was 1.33 m/s and there was left atrium volume indexed of 27.9 mL/m^2^( Figure 2 ). All measurements were within the normal limits, setting up a supernormal mitral flow pattern often found in youth and athletes.

**Figure 2:** – *A) mitral annular tissue Doppler; B) apical four-chamber view on 2D echo bidimensional; C) continuous Doppler of tricuspid regurgitation; D) apical two-chamber view on 2D echo. LAA: left atrial appendage; RA: right atrium; LA: left atrium; E’L: lateral e’ velocity; E’S: septal e’ velocity; RV: right ventricle; LV: left ventricle; Vol AE: left atrium indexed volume.*

Example 2: 48-year-old male bodybuilder and runner. Initial 3D echo study did not show any significant abnormalities. The diastolic evaluation showed an E/A ratio of 1.12, a septal and lateral e’ velocity of 0.05 and 0.07 m/s, respectively, an E/e’ ratio of 10.3, indexed left atrium volume of 17.9 mL/m^2^and tricuspid regurgitation peak velocity of 2 m/s ( Figure 3 ). After analysis of these data, according to the 2016 guideline, only one criterium, out of four, was not normal (mitral septal and lateral annulus velocity), which should lead to a normal diastolic function.

**Figure 3:** – *A) pulsed Doppler curve of mitral valve; B) tissue Doppler curve of mitral lateral annulus. E/A VM: mitral valve E/A ratio; LAEDVindex(A-L): left atrium indexed volume.*

However, attention was drawn to abnormal mitral annulus velocity in an asymptomatic athlete. After a more careful anamnesis, the patient reported that he was on anabolic androgenic steroids (testosterone propionate 30 mg, testosterone phenpropionate 60 mg, testosterone isocaproate 60 mg, testosterone decanoate 100 mg – Durateston®). After evaluation of the myocardial deformation with speckle tracking, we found an abnormal global longitudinal strain value of -15.4%. ( Figure 4 ). This finding completely modifies the diastolic function analysis on this patient. Documented systolic disfunction lead to the second algorithm of 2016 guideline (patients with depressed LVEFs and patients with myocardial disease and normal LVEF after consideration of clinical and other 2D data). Such a low strain value points to some degree of myocardial impairment caused by the steroids, compromising both systolic and diastolic functions. According to this guideline, we should not expect to have systolic disfunction without, at least, some degree of diastolic disfunction, due to the intricated relationship between them. This has not been a new concept. Since 2008 Lester et al.^7^ reported that: “traditionally, parameters of diastolic function have been derived from Doppler and those of systolic function from two-dimensional variables. This may create the illusion that individuals have isolated diastolic dysfunction”. Thus, instead of normal diastolic function, according to the 2016 guideline, this athlete already has mild diastolic disfunction.

**Figure 4:** – *Quantification of myocardial strain by speckle tracking.GLS: global longitudinal strain*

Extremely active people and competitive athlete populations are increasing day by day.^8^ Recent reports extrapolate the causes of cardiac remodeling induced by exercises beyond the ventricular structure, including now changes in diastolic function,^9^ right ventricle morphology,^8^ and left atrium structure.^10 - 12^

All forms of vigorous physical exercise, whether in professional athletes or in highly active people, involve a combination of static and dynamic exercises. Static and dynamic refer to the skeletal muscular activity pattern and its consequence in the cardiovascular system. Static activity is characterized by vigorous short contractions of certain muscular groups. During pure static activity events (or predominantly), like in weightlifting/throwers, we find an acute increase in vascular resistance and blood pressure. The main purpose of cardiovascular system in these athletes is to preserve the cardiac output in face of the sudden and exaggerated increase in afterload. In contrast, dynamic exercises (endurance) are characterized by repetitive contractions and relaxations, often rhythmic, of big muscular groups that require an increase in the oxidative metabolism. The dynamic activity intensity can be quantified by the oxygen consumption (VO_2_). The primary response of the cardiovascular system to dynamic exercise is to increase the cardiac output to ensure the arrival of nutrients to the active muscular bed. Increase of cardiac output is reached by increasing both stroke volume and heart rate and decreasing peripheral vascular resistance.

Diastolic function in athletes and in highly active people should be normal or increased, and any pieces of diastolic disfunction evidence should alert us for any pathology.^13^ Large metanalysis data pointed that exercises promote an increase in diastolic function through the combination of a more effective early diastolic relaxation and increased ventricular compliance.^14^ The type of physical activity is also related to the changes observed in athletes’ diastolic function. Dynamic exercises lead to a more effective ventricular relaxation besides biventricular enlargement, while static exercises may be related to a certain degree of diastolic disfunction,^15^ which usually happens with an increase in wall thickness and left ventricular concentric hypertrophy.

Therefore, it is essential while evaluating ventricular function in athletes, whether they are professionals, amateurs or only “weekend players,” that we use all available tools in the echocardiographic arsenal. Ejection fraction should always be quantified by 3D echo, and evaluation of myocardial deformation (strain measurement) should be taken with the speckle tracking technique. Strain quantification can show incipient impairment in systolic function much earlier than any change in ejection fraction or 2D echocardiographic contractile abnormality could be verified. Routine evaluation of myocardial deformation would allow the detection of some underlying myocardial injury in this population. In addition, a comprehensive analysis of diastolic function must be done according to the recent guideline.

It is quite common to find athletes on formulas and anabolic androgenic steroids without any prescription or medical advice, and a complete echocardiographic evaluation could detect early ventricular systolic and diastolic disfunction, thus allowing correct treatment to avoid any larger myocardial damage.
